# Ultrasensitive Assays Detect Different Conformations of Plasma β Amyloids

**DOI:** 10.1021/acsomega.4c10879

**Published:** 2025-02-11

**Authors:** Chia-Yu Li, Ling-Yun Fan, Chin-Hsien Lin, Chaur-Jong Hu, Ming-Jang Chiu

**Affiliations:** † Department of Neurology, National Taiwan University Hospital, College of Medicine, National Taiwan University, Taipei 100, Taiwan; ‡ Departments of Neurology, National Taiwan University Hospital Bei-Hu Branch, Taipei 108, Taiwan; § Institute of Molecular Medicine, College of Medicine, National Taiwan University, Taipei 100, Taiwan; ∥ Department of Biomedical Engineering, National Taiwan University, Taipei 106, Taiwan; ⊥ Taipei Neuroscience Institute, Taipei Medical University, New Taipei City, 235 Taiwan; # Department of Neurology and Dementia Center, Taipei Medical University-Shuang Ho Hospital, New Taipei City 235, Taiwan; ∇ Department of Neurology, School of Medicine, College of Medicine, Taipei Medical University, Taipei 110, Taiwan

## Abstract

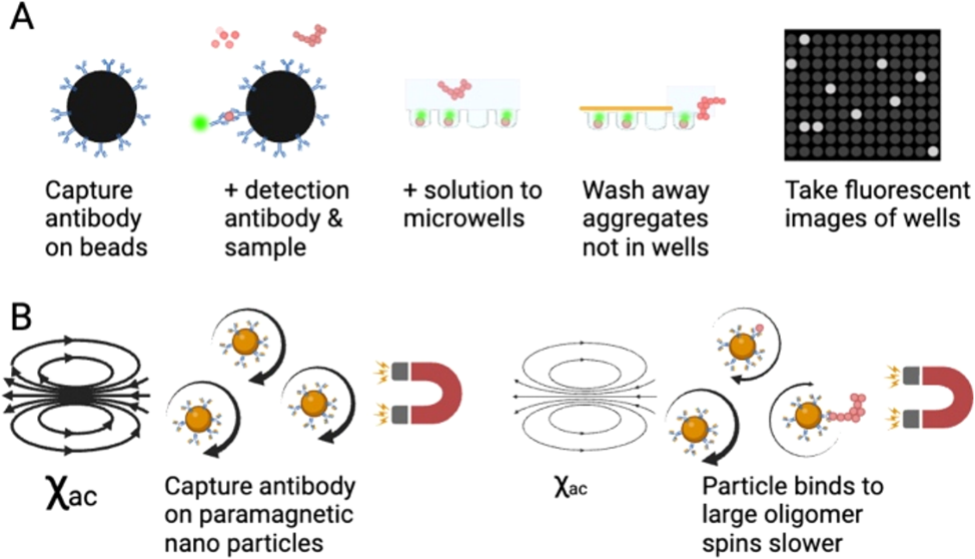

With the developments of ultrasensitive technologies such as immunomagnetic reduction (IMR) assay, single molecule array (SIMOA) assay, electrochemiluminescence immunoassay (ECLIA), the assay of blood-based amyloid 1–42 (Aβ_1–42_) becomes possible. However, the changes in measured plasma Aβ_1–42_ concentrations in Alzheimer’s disease (AD) compared to cognitively unimpaired subjects (CU) are inconsistent. A possible reason for the inconsistency regarding various conformations of Aβ_1–42_ in plasma is explored in this study. Three samples with equal amounts of Aβ_1–42_ but different proportions of monomers and oligomers of Aβ_1–42_ were prepared. The Aβ_1–42_ composition of monomers and oligomers in samples was analyzed with Western blot. Identically diluted versions of these three samples were assayed with IMR and SIMOA for Aβ_1–42_ concentrations. The three diluted samples showed similar levels of Aβ_1–42_ assayed with IMR, whereas much lower levels for samples with more oligomers assayed with SIOMA. The results imply that IMR detects both monomers and oligomers of Aβ_1–42_. The measured levels of Aβ_1–42_ are independent of the proportions of monomer or oligomer Aβ_1–42_ but depend on the total amounts of Aβ_1–42_. In the case of SIMOA, monomers of Aβ_1–42_ are the primary target measured. By comparing Aβ_1–42_ concentrations of the plasma using IMR and SIMOA, the significant difference in plasma Aβ_1–42_ levels using IMR in AD compared to CU is mainly due to the formations of oligomeric Aβ_1–42_. Therefore, if the target molecules are monomers of Aβ_1–42_, SIMOA is the method of choice. Still, if the target molecules should include monomers, small and large oligomers, IMR would be an optimal consideration. In the future, the clinical implications of the proportion of oligomeric Aβ_1–42_ need to be elucidated.

## Introduction

The deposition of β-amyloid in the brain is one of the pathological hallmarks of Alzheimer’s disease (AD).^1−5^ The Amyloid cascade hypothesis became the dominant conceptual theorem for the pathogenesis of AD.^6−8^ Recent advances in Alzheimer’s disease-modifying therapy targeting the removal of amyloid fibrils and plaques support the hypothesis.^5−9^ β-amyloid is a peptide family with 36 to 43 amino acids.^9−12^ β-amyloid 1–42 (Aβ_1–42_) is the most neurotoxic peptide, which can induce mitochondrial dysfunction, oxidative stress, degradation of synaptic proteins, and, consequently, neuronal death.^13−17^ Thus, Aβ_1–42_ plays a crucial role in Alzheimer’s disease (AD). Clinically, it is not only a target for diagnosis but also therapy.

Currently, assays of CSF Aβ_1–42_ concentration and brain amyloid imaging using positron emission tomography (PET) are considered pathological evidence for AD.^18−21^ Decreased levels of CSF Aβ_1–42_ and increased deposition of amyloid plaques in AD compared to cognitive unimpaired subjects (CU) are now biological markers for diagnosing preclinical and clinical AD.^13,22−25^ On the other hand, the relative invasiveness of lumbar puncture and the high costs of amyloid PET prevent them from being used as a first-line screening tool in clinics. An unmet demand to assay Aβ_1–42_ in the body fluid such as blood is urgent.

With the development of ultrasensitive assay technology, precise assays of Aβ_1–42_ in human plasma became feasible.^26,27^ Plasma Aβ_1–42_ levels between AD and CU could differ significantly.^27−32^ However, inconsistency was found among various assay technologies. For example, lower levels of plasma Aβ_1–42_ in AD were revealed by using the single molecular array (SIMOA) assay.^27,28^ The plasma Aβ_1–42_ level was elevated in AD using the immunomagnetic reduction (IMR) assay.^29−32^ Such contradiction puzzles researchers and physicians.

The SIMOA assay, developed and commercialized by Quanterix Inc., uses antibody-coated paramagnetic beads to capture protein aggregates. The beads are then incubated with a biotinylated detector antibody; an immunocomplex is formed with streptavidin β-galactosidase capable of generating a fluorescent readout through resorufin β-d-galactopyranoside.^33^ The SIMOA assay is ultrasensitive for low concentrations, as the beads will only be conjugated to one or zero immunocomplexes. The SIMOA assay is an automatic digital readout immunoassay.^34^

The IMR assay, developed and commercialized by MagQu Co. Ltd., utilizes paramagnetic nanoparticles coated with hydrophilic surfactants and capture antibodies. Under external multiple AC magnetic fields, paramagnetic nanoparticles oscillate; the solution shows a magnetic property called magnetic susceptibility Xac. Due to the association, nanoparticles become larger and consequently oscillate slower (Figure 4). It takes a superconducting-quantum-interference-device (SQUID) AC magnetosusceptometer to read out the susceptibility Xac changes.^26,35^ The IMR assay is ultrasensitive and free from optical interferences from plasma contents or treatment substances in optical density-dependent enzyme-linked immunosorbent assay (ELISA).^36^

In the brain and plasma, soluble Aβ_1–42_ exists in versatile conformations, such as monomers, dimers, oligomers, etc.^37−40^ In AD, Aβ_1–42_ oligomers become dominant compared to monomers.^41−43^ Different assay technologies might target specific Aβ_1–42_ conformations due to antibody selectivity or assay process. We proposed that conformation specificity may account for the contradiction in plasma Aβ_1–42_ levels of AD occurring between assay technologies. We aimed to investigate the target conformations or their combination of Aβ_1–42_ using IMR and SIMOA assays.

## Results

**Figure 1:**
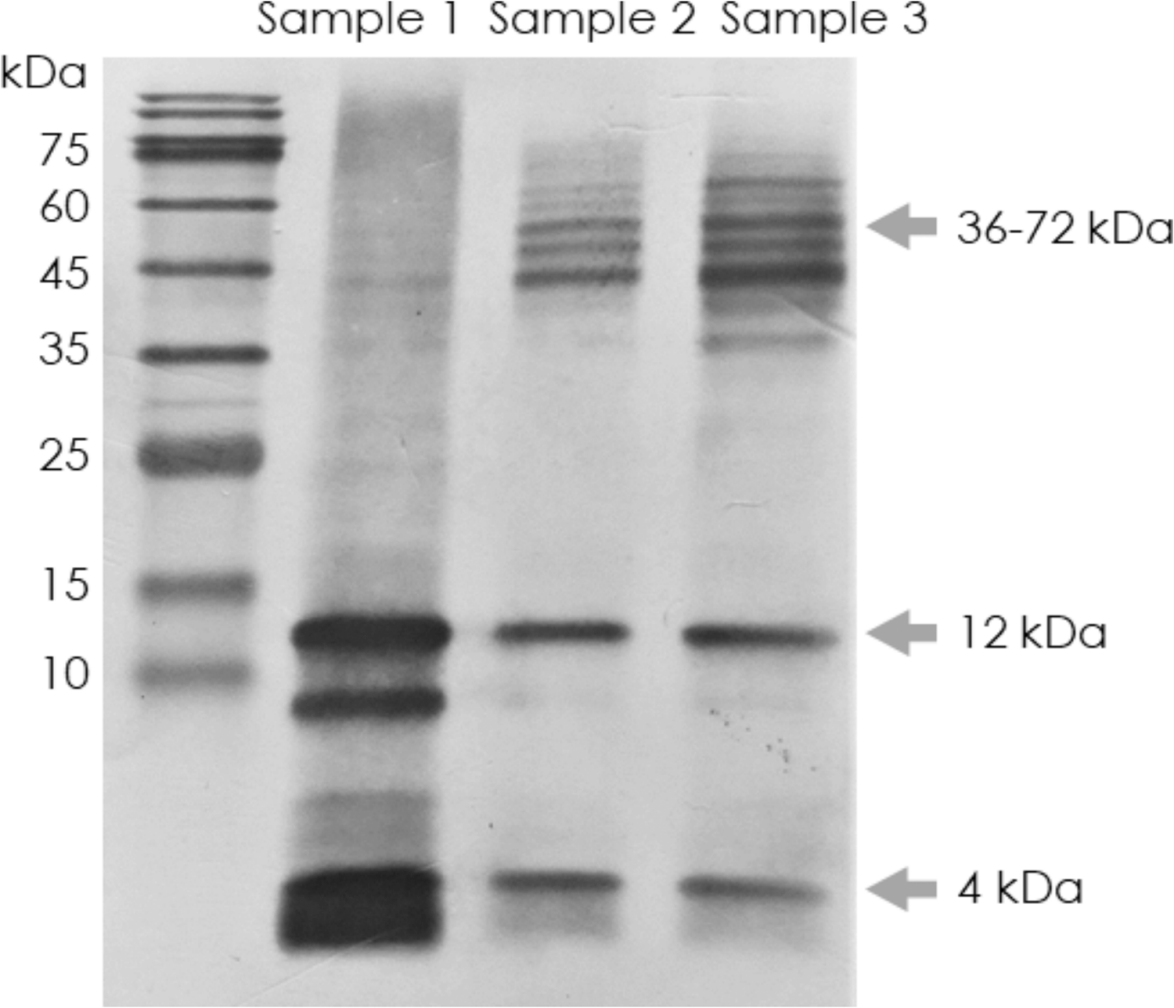
Detection of Aβ_1–42_ monomer-rich and synthetic Aβ_1–42_ oligomer-rich samples were detected by Western blotting. Lane 1: Aβ_1–42_ monomer-rich sample (Sample 1); Lanes 2 and 3: Aβ_1–42_ oligomer-rich samples (Samples 2, 3).

To investigate the types of Aβ_1–42_ detected by two ultrasensitive methods, we utilized a synthetic approach to generate three samples with different ratios of monomer and oligomer. Western blot (WB) was used to verify whether different monomer and oligomer ratios were produced. Figure 1 reveals that the antibody (ab34376, Sigma, 1:1,000) used here can bind with monomers and also oligomers of Aβ_1–42_. It shows the WB results for Samples 1, 2, and 3. The label for four kDa corresponds to the monomer of Aβ_1–42_. The main constituents of sample 1 are monomers (4 kDa), dimers (8 kDa), and trimers (12 kDa). In Sample 2, oligomers of Aβ_1–42_ with molecular weights of 36–72 kDa are found in addition to monomers to trimers. The oligomerization of Aβ_1–42_ was much more enhanced in Sample 3, where the 36–72 kDa are the main components.

**Figure 2:**
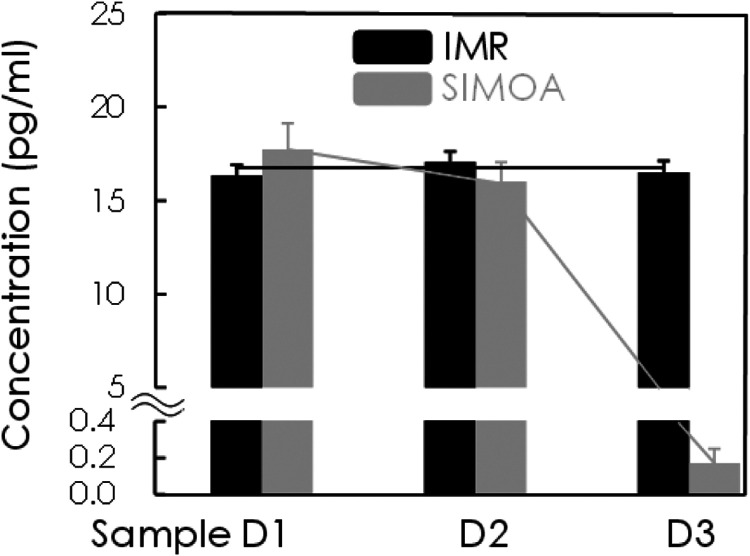
Measured Aβ_1–42_ concentrations in d-Samples 1, 2, and 3 using IMR and SIMOA assays.

Notably, the total Aβ_1–42_ in the three samples was identical, 2 g/mL. Thus, the proportions of monomers and oligomers of Aβ_1–42_ were different among Samples 1, 2, and 3. Using the above samples, we further explored the types of Aβ_1–42_ recognized by IMR and SIMOA. The concentrations of Aβ_1–42_ in diluted Samples 1, 2, and 3 (hereafter designated as d-Samples 1, 2, and 3) were assayed with both IMR and SIMOA assays. The error bars in measured Aβ_1–42_ concentrations are contributed from duplicated measurements (Figure 2, black and gray bars). The IMR assay measured Aβ_1–42_ concentrations for d-Samples 1, 2, and 3 are 16.36 ± 1.78, 17.09 ± 1.45, and 16.51 ± 0.88 pg/mL, respectively (Figure 2, black bars). One-way analysis of variance (ANOVA) showed no significant difference between d-Samples (1, 2, and 3) concentrations measured by IMR assay (*p* = 0.8701). This result indicates that IMR measures the total amount of multiple forms of Aβ_1–42_ and is independent of monomer and oligomer.

The concentrations of Aβ_1–42_ in d-Sample 1, 2, and 3 were also assayed with SIMOA assay. (Figure 2, gray bars). The Aβ_1–42_ concentrations for d-Samples 1, 2, and 3 were 17.75 ± 1.37, 16.03 ± 1.04, and 0.17 ± 0.08 pg/mL, respectively. One-way ANOVA showed significant differences between d-Samples (*p* = 0.0007). The sample having higher proportions of oligomers of Aβ_1–42_ shows lower levels of SIMOA-measured concentrations for Aβ_1–42_. For d-Sample 3, the SIMOA-measured Aβ_1–42_ concentration is much lower, i.e., 0.17 ± 0.08 pg/mL, than their IMR-measured counterparts.

**Figure 3:**
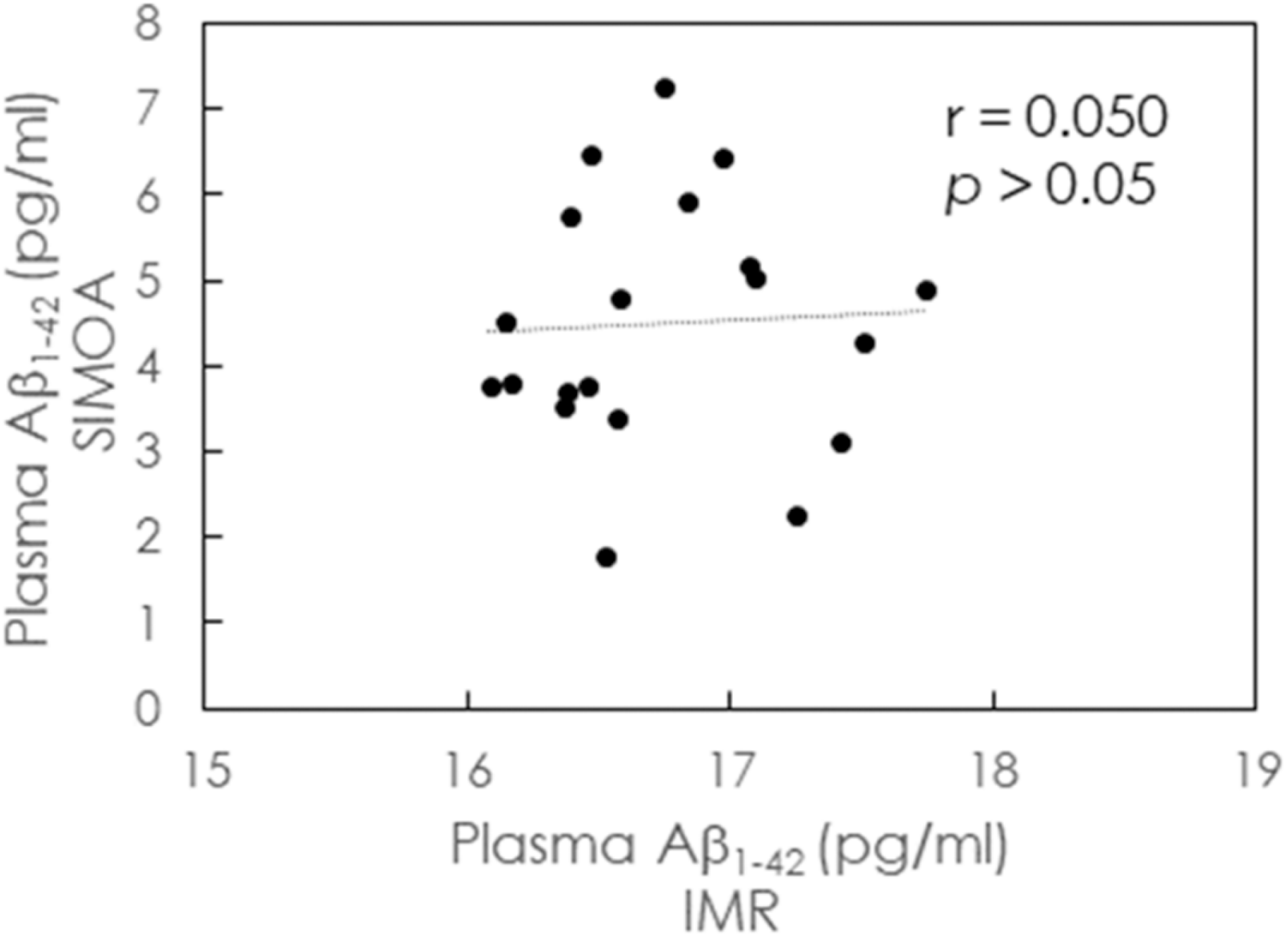
No significant correlation of plasma Aβ_1–42_ concentrations between IMR and SIMOA measurements.

To further confirm whether there is a difference in concentration between IMR and SIMOA in clinical samples due to different aggregate types of Aβ_1–42_. Using IMR and SIOMA assays, the measured Aβ_1–42_ concentrations in human plasma were 16.71 ± 0.49 and 5.18 ± 3.48 pg/mL, respectively. Pearson correlation analysis of the two measurements (*r* = −0.254, *p* > 0.05; dashed line in Figure 3) did not show a significant between-measurement correlation.

It was found that the AD showed significantly higher plasma Aβ_1–42_ levels (17.10 ± 0.40 pg/mL) as compared to that in CU (16.35 ± 0.20 pg/mL, *p* < 0.0001) in the case of IMR assay. However, in the case of the SIMOA assay, the measured plasma Aβ_1–42_ concentrations in AD (4.81 ± 0.40 pg/mL) were higher but not significantly different from those in CU (4.16 ± 1.31 pg/mL, *p* > 0.05).

## Discussion

Although the three samples (Samples 1, 2, and 3) had the same amount of Aβ_1–42_ when prepared, the proportions of monomers and oligomers are different, as evidenced by the Western blot shown in Figure 1.

We found that all three samples showed equivalent levels of Aβ_1–42_ measured with IMR assay (Figure 2). The mechanism of IMR assay could explain this. The reagent of the IMR assay consists of antibody-functionalized magnetic nanoparticles dispersed in a phosphate-buffered saline (PBS) solution. An IMR analyzer applied alternative-current (AC) magnetic fields to oscillate each magnetic nanoparticle; the resultant magnetic response of the reagent to the applied AC magnetic fields is referred to as AC magnetic susceptibility χ_ac_.^35,44^ Once antibody-functionalized magnetic nanoparticles bind with target molecules, the bound nanoparticles grow in size and mass, which results in a slowdown of the spin speed and consequent reduction in the χ_ac_ of solution; this is how the immunomagnetic reduction was named after. In IMR, a single antibody (primary antibody) is utilized. It does not need a secondary antibody. Thus, IMR is referred to as a single antibody assay. Only one epitope is required to join the target molecule in this study, Aβ_1–42_. In this work, the antibody (ab34376, Abcam) is against the C-terminal of Aβ_1–42_, i.e., amino acids 37–42. Bands in Figure 1 contributed from dimers or oligomers are clear. This suggests that the C-terminal of Aβ_1–42_ is exposed not only to monomers but also to oligomers. The exposure of the C-terminal of Aβ_1–42_ in oligomers has also been evidenced in published reports.^45−48^ Thus, all monomers, dimers, and oligomers of Aβ_1–42_ can be bound with antibodies on magnetic nanoparticles in the IMR reagent. The larger the oligomers bound, the more reduction of solution χ_ac_. The amount of the χ_ac_ reduction was transformed to the concentration of the Aβ_1–42_. Thus, IMR assays total Aβ_1–42_. This elaborates why IMR-measured Aβ_1–42_ concentrations are relatively independent of the proportions between monomers and oligomers. IMR assays can detect Aβ_1–42_, covering monomers and oligomers of different sizes ranging from 4–72 kDa (monomers, dimers, trimers to 9–18mers).

**Figure 4:**
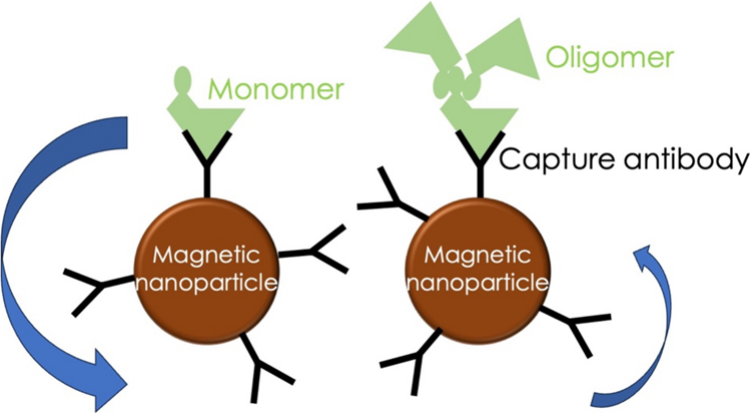
Schematic diagram shows how magnetic nanoparticles bind to monomers and oligomers in an immunomagnetic reduction assay. The magnetic particle binds to a monomer is smaller in size and lower in mass, thus having a higher spin velocity during oscillation. The magnetic particle binds to an oligomer is larger in size and higher in mass, consequently having a lower spin velocity during oscillation. The IMR analyzer applies an alternative current magnetic field to induce oscillation of all magnetic particles in the solution.

On the other hand, the SIMOA-measured concentrations of Aβ_1–42_ are speculated to be dominantly Aβ_1–42_ monomers or possibly dimers and trimers. As shown in Figures 1 and2, IMR assays total Aβ_1–42_, whereas monomers and small oligomers (dimers or trimers) of Aβ_1–42_ are dominant constituents in SIMOA. This might explain why the measured concentrations of Aβ_1–42_ do not correlate between IMR and SIOMA (Figure 3).^49^ According to our understanding, discussions on such a difference in the dominant conformations of Aβ_1–42_ between IMR and SIMOA were rare. We want to explore the possible reasons (Figure 4).

In SIMOA, carboxylated paramagnetic beads (2.7 μm) were conjugated with purified monoclonal β amyloid antibody (capture antibody), followed by digital enzyme-linked immunosorbent assay (ELISA) to quantify Aβ_1–42_.^33,50^ The detector antibody used an Aβ_1–42_ recombinant rabbit monoclonal antibody. Digital ELISA is a utility of sandwiched assay involving capture antibody and detector antibody binding with two individual epitopes of a target molecule. For example, the capture antibody binds with the C-terminal of Aβ_1–42_, and the detector antibody binds with the other epitope of Aβ_1–42_. Once Aβ_1–42_ monomers aggregate to form dimers or oligomers, one of the binding epitopes for the detector antibody is easily blocked, or the structure of the epitope is modified, preventing aggregated Aβ_1–42_ molecules from associating with the detector antibody. Thus, aggregated Aβ_1–42_ molecules would not be visible on the fluorescence images of the autoimmunoassay analyzer.

On the other hand, both epitopes of monomers are readily exposed to capture antibody and the detector antibody in digital ELISA. Thus, monomers of Aβ_1–42_ are easily detected by the autoimmunoassay analyzer. Upon incubation of microbeads with the target molecules, the detector reagent (including the reagents used to generate fluorophores) is added, and beads are then distributed over an array of microwells; each one is capable of confining a single microbead because of the very low volume (femtoliter) of the microwells. In addition, when the target protein aggregates into oligomers or forms a complex with other proteins, the magnetic beads may not be able to smoothly fall into a small-volume well aiming to capture a single molecule on the plate because of their large size in the SIMOA system. It is removed in subsequent wash steps, thus underestimating the concentration of the target protein, especially for those large oligomers.

In Table 1, the levels of Aβ_1–42_ in human plasma measured by IMR significantly increase in AD compared to CU, which agrees with previous studies utilizing IMR.^29−32^ The significantly elevated levels of measured Aβ_1–42_ using IMR reveal an increase in the total amounts of Aβ_1–42_ in human plasma in AD compared to CU. It is rational to conclude that the increase in the total amounts of Aβ_1–42_ in human plasma in AD is contributed by oligomeric forms of Aβ_1–42_ but not monomers.

**Table 1:** Age, Education Year, CDR, and Plasma Aβ_1-42_ Concentrations Measured by IMR and SIMOA in CU and AD^a^

groups	CU	AD
number (female%)	10 (60%)	10 (80%)
age (years)	56.5 ± 7.8	82.5 ± 4.4***
education (years)	14.8 ± 2.4	8.4 ± 4.4***
CDR	0	1.10 ± 0.91***
Aβ_1–42_ (pg/mL) - IMR	16.37 ± 0.18	17.10 ± 0.40***
Aβ_1–42_ (pg/mL) - SIMOA	4.16 ± 1.31	4.81 ± 0.40

a CDR: clinical dementia rating; AD subjects with Alzheimer’s disease; CU: cognitively unimpaired subjects; *** *p* < 0.0001 compared to CU; IMR: immunomagnetic reduction assay; SIMOA: single molecular array assay.

As shown in Figure 2, the dominant conformations to the measured concentrations of Aβ_1–42_ using SIMOA are monomers or probably small oligomers. The insignificant difference in measured Aβ_1–42_ concentrations in human plasma using SIMOA between AD and CU implies that Aβ_1–42_ monomers remain unchanged or even decreased between AD and CU. The equivalent levels of measured Aβ_1–42_ in human plasma using SIMOA between AD and CU were also reported by other groups^51,52^ It is reasonable to deduce that the increase in the total amounts of plasma Aβ_1–42_ in AD could be possibly due to the rise in the quantities of oligomers of Aβ_1–42_. Some other groups showed decreased levels of Aβ_1–42_ in human plasma using SIMOA in AD compared to CU.^27,28^ This suggests that amounts of monomers of Aβ_1–42_ in AD might be reduced, not equivalent, compared to CU. All the results indicate that the amounts of plasma monomers of Aβ_1–42_ could be either unchanged or slightly lower in AD than in CU. The oligomers of Aβ_1–42_ would be the critical conformation in human plasma in AD.

Due to their neurotoxicity, aggregates of oligomeric Aβ_1–42_ play a role in the pathogenesis of AD.^41−43,53,54^ The mechanisms for forming soluble oligomers of Aβ_1–42_ are under discussion. Most researchers believe the main factor is the hydrophobic nature.^55−57^ The more oligomeric form Aβ_1–42_, the more neuronal damage, which leads to more rapid cognitive deterioration. This suggests the use of plasma oligomers of Aβ_1–42_ as an indicator of the occurrence of cognitive impairment.

Several groups used IMR to assay plasma Aβ_1–42_ and found the elevations of plasma Aβ_1–42_ concentrations in aMCI and AD compared to CU.^29−32^ As deduced, the elevations of plasma Aβ_1–42_ assayed with IMR would be mainly due to the increase in the soluble oligomers of Aβ_1–42_. These published results prove cognitive deterioration in subjects with higher levels of Aβ_1–42_ soluble oligomers in human plasma. In addition, Tsai et al. and Chen et al. independently demonstrated that the patients of amnestic mild cognitive impairment (aMCI) having higher levels of plasma Aβ_1–42_ assayed with IMR at baseline were at high risk for cognitive decline.^58,59^ This could imply that the levels of soluble oligomers of A Aβ_1–42_ in plasma played a predicting index for cognitive decline in aMCI. Nevertheless, not only Alzheimer’s disease can show increased amyloid deposits in the brain and other body fluids, but some subjects of Lewy body dementia and Parkinson’s disease dementia (PDD) can have increased brain amyloid deposits.^60^ This can also be due to mixed pathology.^61^ Previous studies also found that some PDD subjects might have increased blood amyloid Aβ1–42.^62,63^ In this study, we only included those subjects with very mild (CDR = 0.5) or mild (CDR = 1) dementia due to AD, and none of them revealed Parkinsonian features. Clinical correlation must always be taken carefully in interpreting the blood amyloid level Aβ1–42, considering the concept of mixed pathology commonness.

There are several limitations to this study. First, the diluted version of Samples 1, 2, and 3 may not have identical proportions between monomers and oligomers as in their undiluted counterparts. Theoretically, there is always the possibility of a two-way dynamics of conformation change between monomers and oligomers. However, this study used a relatively high concentration (400 μM) of Aβ_1–42_ at 37 °C to obtain an efficient oligomeric formation dozens of million times higher than the diluted samples (20 pg/mL). Therefore, there is minimal opportunity for the monomers to form oligomers in such a low-concentration solution and unfavorable thermodynamic condition. On the other hand, though possible, the dissociation of oligomers to monomers takes fibril surfaces to catalyze the oligomer dissociating process, which is not the scenario in our experiment.^64^ Second, in the human plasma study, we only observed significant between-group differences with the IMR measurement but not SIMOA. This could suffer from the small sample size of our research, but again, the total amount of Aβ_1–42_ consisting of monomers and oligomers should have an adequate effect size to differentiate AD from CU. Third, we did not perform further correlation analysis of the clinical symptoms due to the small sample size of patients covering only mild and very mild dementia and controls. Lastly, this is a cross-sectional study. Therefore, we cannot provide information about the link between our test results and the disease progression. However, this critical issue is worth exploring in the future.

## Conclusions

IMR simultaneously detects monomers and oligomers of Aβ_1–42_. The measured Aβ_1–42_ level using IMR is independent of the proportions between monomers and oligomers but depends on the total amounts of Aβ_1–42_. In SIMOA, monomers of Aβ_1–42_ are the main constituents measured. By comparing Aβ_1–42_ plasma concentrations using IMR and SIMOA, the significant difference in plasma Aβ_1–42_ levels using IMR in AD compared to CU is mainly due to oligomeric Aβ_1–42_ formations. Using different ultrasensitive measurements of the plasma Aβ_1–42_, one must consider the conformation of the target molecules. Therefore, if the target molecules are Aβ_1–42_ monomers, SIMOA is the method of choice. Still, if the target molecules should include monomers, small and large oligomers, IMR would be an optimal consideration. In the future, the clinical implications of the oligomeric Aβ_1–42_ proportion need to be elucidated.

## Materials and Methods

In the first step, we prepared samples with equal amounts of Aβ_1–42_ with different proportions of monomers and oligomers. The Aβ_1–42_ proportion in each sample is examined with Western blot. IMR and SIMOA were used to assay the samples’ Aβ_1–42_ concentrations. Besides the Aβ_1–42_ concentrations in human plasma, ten samples of CU and ten patients with AD were assayed with IMR and SIMOA to investigate the relation between IMR-measured and SIMOA-measured concentrations of Aβ_1–42_.

### Sample Preparation

The Aβ_1–42_ peptide (H-1368, Bachem) was dissolved using 100% 1,1,1,3,3,3 hexafluoro-2-propanol (HFIP) at a 6 mg/mL concentration. The Aβ_1–42_ solution was incubated at 37 °C for 1.5 h to dissolve completely, and HFIP was removed using Speedvac. Aβ_1–42_ was resuspended in dimethyl sulfoxide (DMSO) at 5 mM and sonicated for 20 s, denoted as Sample 1. In Sample 1, monomers (4 kDa) were supposed to be the main component. The HFIP-treated Aβ_1–42_ was diluted to 400 μM with phosphate-buffered saline (PBS) containing 1/10 volume sodium dodecyl sulfate solution (SDS) (2% in H_2_O). After incubation at 37 °C for 6 h, the intermediate oligomers with 16/20 kDa Aβ_1–42_ were formed, denoted as Sample 2. The 38/48 kDa Aβ_1–42_ oligomers were prepared by dilution with triplet volumes of H_2_O and incubated at 37 °C for 18 h, denoted as Sample 3.^65^

### Western Blot

Equal amounts of Aβ_1–42_ solutions (2 μg) were prepared for Western blot studies. The poly(vinylidene fluoride) (PVDF) membranes were incubated in PBS with 5% bovine serum albumin for 1 h at room temperature and then incubated in PBS with the appropriate primary or secondary antibody. Primary antibodies used for Western blotting included rabbit anti-Aβ_1–42_ (ab34376, Sigma, 1:1,000). Secondary antibodies conjugated with alkaline phosphatase (A3687, Sigma) were used at a dilution of 1:10,000. Finally, the membranes were visualized using BCIP/NBT substrate solution (B3804, Sigma).

### Aβ_1–42_ Concentration Measurements

Samples 1, 2, and 3 were diluted to about 20 pg/mL with the identical procedure and proportion, which resulted in diluted Samples 1, 2, and 3 (denoted as d-Samples 1, 2, and 3). Aβ_1–42_ d-Samples 1, 2, and 3 concentrations were measured using IMR and SIMOA, respectively. In IMR, the reagent (MF-AB2–0060, MagQu) and the analyzer (XacPro-S, MagQu) were utilized to assay Aβ_1–42_ in d-Samples 1, 2, and 3. Aβ_1–42_ in d-Samples 1, 2, and 3 were detected in SIMOA using the Aβ_1–42_ 2.0 kit (101644, Quanterix) and the digital autoimmunoassay analyzer (HD-1, Quanterix). Duplicated measurements of Aβ_1–42_ concentrations were done for each sample. The mean value and the standard deviation of the duplicated measurements were reported.

### Preparation of Human Plasma

Twenty subjects were recruited in this study; 10 had normal cognition (6 women), and 10 were people living with AD (Table 1).

The clinical dementia rating (CDR) was 0.52 ± and 0.83 for the AD group. Alzheimer’s disease (AD) was diagnosed according to NIA-AA guidelines.^66^ Ten were CU, and 10 were AD (Table 1).

The study was approved by the ethics committee of the National Taiwan University Hospital (202012267RIND).

All subjects donated a 9 ml nonfasting venous blood sample (K3 EDTA, lavender-top tube) between 10 AM and 2 PM, followed by gently inverting the tube ten times immediately after blood collection. The blood samples were placed at room temperature before centrifugation. They were centrifuged at room temperature within 4.5 h of collection (2500*g* for 15 min). Plasma was aliquoted into cryotubes and stored at −80 °C for less than one year until thawed for measurement.

### Assay of Aβ_1–42_ in Human Plasma

Aβ_1–42_ human plasma concentrations were measured using IMR and SIMOA, respectively. In IMR, the reagent (MF-AB2–0060, MagQu) and the analyzer (XacPro-S, MagQu) were utilized to assay Aβ_1–42_ in human plasma. SIMOA used the Aβ_1–42_ 2.0 kit (101644, Quanterix) and the autoimmunoassay analyzer (HD-1, Quanterix) to measure plasma Aβ_1–42_. Duplicated measurements of Aβ_1–42_ concentrations were done for each sample. The mean value and the standard deviation of the duplicated measurements were reported.

### Statistics

Continuous variables for each measurement are presented as the mean ± standard deviation. The between-sample difference was compared using one-way ANOVA, and significant levels were designated at the p-value <0.05. Pearson correlation analysis examined the relation between IMR- and SIMOA-measured plasma Aβ_1–42_ concentrations. The statistics and graphing were performed with GraphPad Prism Version 11.
